# Canine Splenic Hemangiosarcoma: Biological Behavior, Clinical Challenges and Therapeutic Limitations

**DOI:** 10.3390/ani16050778

**Published:** 2026-03-02

**Authors:** Felisbina Pereira Queiroga, Ana Margarida Marques, Hugo Gregório, Gonçalo N. Petrucci

**Affiliations:** 1 Department of Veterinary Sciences, University of Trás-os-Montes and Alto Douro, 5001-801 Vila Real, Portugal; ana-margarida@live.com; 2 Animal and Veterinary Research Center (CECAV), University of Trás-os-Montes and Alto Douro, 5001-801 Vila Real, Portugal; hugogregvet@hotmail.com (H.G.); goncalopetrucci@gmail.com (G.N.P.); 3 Institute for Research and Advanced Training in Health Sciences and Technologies, Cooperativa de Ensino Superior Politécnico e Universitário (CESPU), 4585-116 Gandra, Portugal; 4 AniCura, CHV Porto, 4100-320 Porto, Portugal; 5 OneVet Group, Hospital Veterinário do Porto, 3020-210 Porto, Portugal; 6 UNIPRO—Oral Pathology and Rehabilitation Research Unit, University Institute of Health Sciences (IUCS), Cooperativa de Ensino Superior Politécnico e Universitário (CESPU), 4585-116 Gandra, Portugal

**Keywords:** canine splenic hemangiosarcoma, vascular tumors, metastasis, diagnosis, chemotherapy, prognosis

## Abstract

Canine splenic hemangiosarcoma is a highly malignant vascular neoplasm and is among the most frequent and clinically relevant splenic tumors in dogs. Its biological behavior is characterized by rapid growth, marked invasiveness, and early metastatic dissemination, contributing to the poor prognosis commonly observed in affected animals. Clinically, splenic hemangiosarcoma often remains subclinical until acute presentation due to splenic rupture and hemoperitoneum, thereby substantially limiting opportunities for early diagnosis and timely therapeutic intervention. Despite advances in diagnostic imaging, surgical techniques, and the use of adjuvant chemotherapy, the impact of current therapeutic approaches on long-term survival remains limited. Splenectomy is primarily palliative for hemorrhage control, and adjuvant doxorubicin-based chemotherapy yields only modest improvements in median survival; alternative approaches (metronomic chemotherapy, immunotherapy, and targeted therapies) have not demonstrated consistent clinical benefit. This review summarizes the biological and pathophysiological features of canine splenic hemangiosarcoma, discusses the main clinical challenges associated with its diagnosis and staging, and critically reviews current therapeutic approaches and their limitations. By integrating biological behavior with clinical and therapeutic evidence, this article highlights the reasons why prognosis remains poor and underscores the need for more effective strategies to improve clinical outcomes in dogs with splenic hemangiosarcoma.

## Simple Summary

Splenic hemangiosarcoma is one of the most aggressive malignancies affecting dogs and frequently remains clinically silent until it precipitates acute intra-abdominal hemorrhage following splenic rupture. Due to the highly metastatic nature of this tumor, microscopic metastases are typically present at the time of diagnosis. Surgical excision combined with systemic chemotherapy represents the current standard of care; however, these interventions generally result in only modest prolongation of survival. Alternative strategies, including immunotherapeutic and targeted approaches, have been explored, but outcomes have been variable and inconsistent. This review summarizes the biological behavior of splenic hemangiosarcoma, outlines the principal diagnostic and therapeutic challenges, and discusses the factors contributing to the persistently poor prognosis despite advances in treatment.

## 1. Introduction

Splenic disease is a frequent clinical finding in dogs and may be identified during routine physical examination or diagnostic imaging. Splenomegaly is a descriptive term that refers to focal or generalized enlargement of the spleen and may result from a wide range of non-neoplastic and neoplastic conditions {Valli, 2016 #15} [1,2,3,4,5]. Focal splenomegaly, commonly presenting as a nodular lesion or splenic mass, is the most frequent presentation in dogs and is often associated with nodular hyperplasia, hematoma, and hemangiosarcoma [2,5]. In contrast, generalized splenomegaly usually reflects diffuse pathological processes, including inflammatory conditions, hyperplasia, hematopoiesis, vascular alterations such as congestion or hyperemia, and infiltration by cells or substances, including neoplastic cells or amyloids [3,4,5].

Splenic neoplasia encompasses a heterogeneous group of tumors ranging from benign to malignant, primary or metastatic, and that can manifest as focal or diffuse splenic enlargement [3,4,5,6]. As one of the main reticuloendothelial organs, the spleen is particularly predisposed to the development of tumors of hematopoietic and vascular origin [7]. Primary splenic neoplasms may arise from endothelial cells, lymphoid tissue, smooth muscle, or connective tissue stroma, whereas secondary splenic involvement may occur through metastatic dissemination from distant sites [3,4,6]. Although the spleen is highly vascularized, metastatic splenic tumors are considered relatively uncommon, likely due to the phagocytic activity of the splenic mononuclear system; nevertheless, lymphoma represents the most frequently reported metastatic neoplasm involving the spleen [1,3,4,8].

Among malignant splenic tumors in dogs, hemangiosarcoma and lymphoma are the most common, typically affecting middle-aged to older animals, with several breeds reported as overrepresented [9]. Canine splenic hemangiosarcoma is a malignant vascular neoplasm with a markedly higher prevalence in dogs compared with other domestic species [10,11]. Although its exact etiology remains unclear, dysregulation of angiogenesis has been implicated in its pathogenesis, including overexpression of pro-angiogenic growth factors and their receptors in canine hemangiosarcoma cell lines [9]. Historically, hemangiosarcoma was believed to originate from peripheral endothelial cells; however, more recent evidence suggests that it may arise from bone marrow progenitor cells that undergo abnormal differentiation and subsequently migrate to peripheral vascular sites [12].

Biologically, splenic hemangiosarcoma exhibits highly aggressive behavior, characterized by rapid growth, local tissue invasion, and early metastatic dissemination [13,14]. Reported metastatic rates range from 67% to nearly 100%, with common metastatic sites including the lungs, heart, liver, omentum, and peritoneum [13,14]. The tumor may remain clinically silent for prolonged periods and often becomes apparent only after rupture of the splenic mass, leading to acute hemoperitoneum and hemodynamic compromise [11,14,15]. Consequently, many dogs present as emergency cases, which limits opportunities for early diagnosis, comprehensive staging, and therapeutic planning.

From a clinical standpoint, splenic hemangiosarcoma poses substantial diagnostic and therapeutic challenges. For clinical staging, while conventional protocols commonly include thoracic radiography, abdominal ultrasonography, and echocardiography, Computed Tomography (CT) of the chest and abdomen is increasingly used when available and may improve lesion detection and surgical planning [14]. Surgical splenectomy remains the cornerstone of initial management and is essential for controlling life-threatening hemorrhage; however, surgery alone is largely palliative and does not address the high likelihood of micrometastatic disease present at diagnosis [14,16].

Adjuvant chemotherapy, most commonly based on doxorubicin protocols, has been widely used in an attempt to improve survival outcomes, yet reported gains in median survival time remain modest [14,16,17,18,19,20]. Several alternative therapeutic approaches, including combination chemotherapy protocols, metronomic chemotherapy, immunotherapy, angiogenesis inhibitors, and targeted therapies, have been investigated, but results are variable [10,14,18,21,22,23] and often inconsistent across studies [24].

Given the aggressive biological behavior of splenic hemangiosarcoma and the limited impact of currently available treatments on long-term survival, a clear understanding of its pathophysiology, clinical presentation, and therapeutic limitations is essential for realistic prognostication and informed clinical decision-making [13]. This review aims to summarize the biological and pathophysiological features of canine splenic hemangiosarcoma, discuss the main clinical challenges associated with its diagnosis and staging, and critically evaluate current therapeutic approaches, with particular emphasis on their limitations and their impact on clinical outcomes.

## 2. Biological and Pathophysiological Features of Canine Splenic Hemangiosarcoma

Canine splenic hemangiosarcoma is a malignant neoplasm characterized by a highly complex and unstable biological behavior, which underlies its aggressive clinical course. The spleen, as a highly vascularized organ and a central component of the reticuloendothelial system, is predisposed to the development of neoplasms of hematopoietic and vascular origin [7]. Among these, hemangiosarcoma represents the most frequent malignant vascular tumor affecting the canine spleen [9,10,25].

For many years, hemangiosarcoma was considered to arise from mature peripheral endothelial cells lining blood vessels. However, accumulating evidence suggests that canine hemangiosarcoma may originate from bone marrow-derived progenitor cells that undergo dysregulated differentiation and subsequently migrate to peripheral vascular sites, including the spleen [12]. This shift in the proposed cellular origin has important biological and therapeutic implications, as these progenitor cells appear to retain stem-like properties, contributing to tumor plasticity, intratumoral heterogeneity, and resistance to conventional cytotoxic therapies. These progenitor cells are thought to differentiate into angioblast-like cells, contributing to the formation of highly abnormal vascular structures that characterize the tumor [11,12].

Angiogenesis plays a central role in the pathogenesis of splenic hemangiosarcoma. Alterations in angiogenesis-regulatory pathways have been identified in canine hemangiosarcoma cell lines, including overexpression of pro-angiogenic growth factors such as vascular endothelial growth factor, fibroblast growth factor, and angiopoietins, as well as their corresponding receptors [9]. Rather than reflecting a controlled neovascular response, this dysregulated angiogenic signaling promotes the formation of fragile, tortuous vascular channels that are structurally and functionally abnormal, predisposing the tumor to hemorrhage, thrombosis, and necrosis [11].

Histologically, splenic hemangiosarcoma is characterized by pleomorphic endothelial cells arranged in irregular vascular spaces and multifocal cavities containing variable amounts of blood and thrombi, often accompanied by extensive areas of hemorrhage and necrosis [13,16]. Marked cellular atypia, a high mitotic index, and poor overall differentiation are common features and reflect the high-grade malignant nature of the tumor [13,16,18]. These histological features mirror the underlying biological instability of the neoplasm and are closely associated with its aggressive clinical behavior, including rapid growth and early metastatic dissemination [14]. Although histological grading systems have been proposed for canine hemangiosarcoma, their reproducibility and independent prognostic value remain inconsistent across studies [13,14,18]. Variability in grading criteria, interobserver differences, and the predominance of retrospective study designs limit their applicability in routine clinical decision-making [13,14]. Consequently, while histopathological evaluation confirms diagnosis and may provide insight into tumor aggressiveness, clinical stage remains the most robust prognostic parameter in practice [14,16,18].

The biological instability of the neoplastic vasculature has important pathophysiological consequences. Tumor-associated blood vessels are frequently disorganized and prone to thrombosis, which compromises blood flow within the tumor mass. This results in ischemia and necrosis of neoplastic tissue, followed by rupture of vascular structures and intratumoral hemorrhage [11,15]. When rupture extends beyond the splenic capsule, acute hemoperitoneum may occur, representing one of the most common and life-threatening clinical manifestations of splenic hemangiosarcoma [14,15].

Metastatic dissemination occurs early in the course of the disease and may proceed through hematogenous spread or local implantation following tumor rupture [13,14,17]. Common metastatic sites include the lungs, liver, omentum, muscle, peritoneum, and heart, with hemangiosarcoma being the most frequent tumor to metastasize to the brain in dogs [13]. The remarkably high metastatic rate, reported to range from 67% to nearly 100%, reflects the intrinsic invasive potential of the neoplastic cells and the permissive vascular environment created by the tumor itself [14,17].

In addition to their role in tumor growth and dissemination, the biological characteristics of hemangiosarcoma contribute to marked intratumoral heterogeneity. Variability in cellular differentiation, vascular organization, and proliferative activity may be observed within the same tumor mass, reflecting the dynamic and unstable nature of the neoplastic process [11,13,14]. This heterogeneity is increasingly recognized as a key factor influencing both disease progression and therapeutic response, as distinct subpopulations of neoplastic cells may differ in their sensitivity to cytotoxic agents, antiangiogenic strategies, and immunomodulatory therapies [10,11,13]. Such biological complexity further complicates disease control and likely contributes to the limited and variable therapeutic outcomes reported in dogs with splenic hemangiosarcoma [13,14].

Beyond aberrant angiogenic signaling, increasing evidence supports the concept that splenic hemangiosarcoma is characterized by significant molecular heterogeneity. Variations in gene expression profiles, signaling pathway activation, and endothelial differentiation status suggest that this tumor does not represent a biologically uniform entity. Dysregulation of pathways involved in angiogenesis, cell survival, and inflammatory signaling, including VEGF, PI3K/AKT, and MAPK cascades, has been described and may contribute to tumor progression and therapeutic resistance [13,14].

In addition, the tumor microenvironment appears to play a critical role in disease aggressiveness. Gene expression profiling studies have demonstrated that canine hemangiosarcoma exhibits transcriptional signatures consistent with inflammatory activation, dysregulated angiogenic signaling, and altered immune-related pathways, supporting the concept that tumor progression is influenced not only by neoplastic endothelial cells but also by surrounding stromal and immune components [11,26]. Splenic hemangiosarcoma is highly vascularized and frequently associated with intratumoral hemorrhage, inflammatory cell infiltration, and thrombotic phenomena, creating a dynamic microenvironment that promotes tumor cell survival and dissemination. Interactions between neoplastic endothelial cells, platelets, immune cells, and stromal elements may facilitate immune evasion and metastatic spread, contributing to therapeutic resistance and disease progression [11,27]. These complex biological interactions likely contribute to the limited and inconsistent response to current therapeutic strategies.

**Figure 1:**
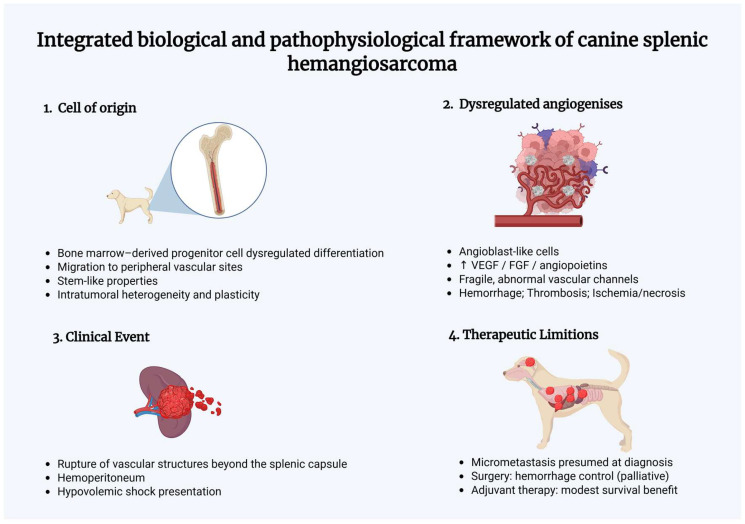
The schematic summarizes key mechanisms driving canine splenic hemangiosarcoma progression and clinical outcome. Tumor initiation is linked to a proposed bone marrow-derived progenitor cell origin and stem-like traits, promoting intratumoral heterogeneity and treatment resistance. Dysregulated angiogenesis (e.g., increased VEGF/FGF/angiopoietin signaling) results in fragile, abnormal vascular channels that predispose to thrombosis and necrosis. These changes favor intratumoral hemorrhage and splenic rupture, frequently leading to hemoperitoneum and acute hypovolemic shock. Early metastatic dissemination is presumed in most cases at diagnosis, explaining why treatment is largely palliative for hemorrhage control and why chemotherapy provides only modest survival benefit.

The integrated biological and pathophysiological mechanisms driving canine splenic hemangiosarcoma, from tumor initiation and dysregulated angiogenesis to rupture-associated hemoperitoneum, early metastatic dissemination, and limited therapeutic efficacy, are summarized in Figure 1.

## 3. Clinical Presentation and Diagnostic Challenges

Canine splenic hemangiosarcoma often presents a significant diagnostic challenge due to its insidious clinical course and nonspecific initial manifestations. In many dogs, the tumor develops silently and remains clinically unapparent until an acute event occurs, most commonly rupture of the splenic mass with subsequent hemoperitoneum [11,14,15]. As a result, a substantial proportion of affected animals are presented in emergency settings with signs of hypovolemic shock, including weakness, lethargy, pale mucous membranes, tachycardia, and abdominal distension [11,15,28].

Clinical signs associated with splenic hemangiosarcoma are frequently related to anemia and blood loss rather than to the tumor mass itself. The abnormal and fragile neoplastic vasculature predisposes to repeated intratumoral hemorrhage and clot formation, which may lead to episodic bleeding into the abdominal cavity [11,28]. Depending on the volume and rate of blood loss, dogs may exhibit acute collapse or intermittent, vague clinical signs that temporarily resolve, as hemorrhaged blood is reabsorbed and erythropoiesis partially compensates for anemia [11,15]. This fluctuating clinical pattern may falsely suggest clinical improvement and contribute to delayed diagnosis or underestimation of disease severity.

Physical examination findings are often nonspecific. Abdominal palpation may reveal splenomegaly or a palpable mass, although this is not consistently detectable, particularly in large or deep-chested dogs [2,5]. Laboratory abnormalities commonly include regenerative anemia and, in some cases, thrombocytopenia, both of which are associated with tumor-related hemorrhage and may have prognostic significance [28,29]. However, these abnormalities lack diagnostic specificity, as they may also be observed in dogs with benign splenic lesions or other causes of hemoperitoneum, limiting their utility as standalone diagnostic indicators.

**Figure 2:**
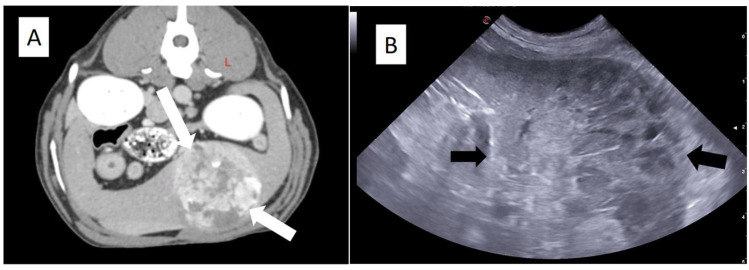
Imaging appearance of canine splenic hemangiosarcoma. (**A**) Contrast-enhanced computed tomography (after intravenous injection of iodinated contrast medium), transverse image showing a large heterogeneous splenic mass (white arrows). (**B**) Abdominal ultrasonography (longitudinal plane) showing a splenic mass (black arrows) with a heterogeneous echotexture and irregular architecture, consistent with a hemangiosarcoma (confirmed with histology after splenectomy). In both images there is no hemoperitoneum (images courtesy of Onevet Porto Veterinary Hospital).

Diagnostic imaging plays a central role in the evaluation of dogs with suspected splenic hemangiosarcoma (Figure 2). Abdominal ultrasonography is widely used to identify splenic masses, assess splenic architecture, and detect free abdominal fluid [2,3,14]. Nevertheless, ultrasonographic features alone cannot reliably differentiate malignant from benign splenic lesions, as hematomas, nodular hyperplasia, and hemangiosarcoma may share similar echogenic patterns, lesion margins, and internal heterogeneity [6,30]. Although certain imaging characteristics may raise suspicion for malignancy, considerable overlap persists, resulting in limited preoperative diagnostic accuracy. Advanced imaging modalities, such as computed tomography or magnetic resonance imaging, may provide improved anatomical detail and facilitate surgical planning, particularly in complex or atypical cases [25]. However, their availability remains limited in many clinical settings, and, importantly, these techniques do not allow definitive distinction between benign and malignant splenic masses, thereby restricting their impact on preoperative decision-making.

Definitive diagnosis of splenic hemangiosarcoma requires histopathological examination of splenic tissue obtained following splenectomy [13,14]. Fine-needle aspiration is generally discouraged due to the high risk of hemorrhage and the limited diagnostic yield associated with this technique in vascular tumors [3,14]. Consequently, diagnosis is often established only after surgical removal of the spleen, frequently performed as an emergency procedure to control life-threatening bleeding.

An additional diagnostic challenge in dogs with splenic hemangiosarcoma is the current lack of reliable noninvasive biomarkers capable of differentiating malignant from benign splenic lesions prior to surgery [11,14]. Although clinical findings, laboratory abnormalities, and imaging features may raise suspicion for malignancy, considerable overlap exists between hemangiosarcoma and benign conditions such as hematoma or nodular hyperplasia [3,15,30]. As a result, preoperative prognostic counseling is often imprecise, and definitive diagnostic and therapeutic decisions are frequently deferred until histopathological results are available following splenectomy [14,18]. This diagnostic uncertainty complicates clinical decision-making and may influence owner expectations at the time of presentation [14,15].

Comprehensive clinical staging is essential for prognostication and therapeutic planning, although it is often constrained by the patient’s clinical instability at presentation. Staging typically includes thoracic radiographs to evaluate pulmonary metastasis, abdominal imaging to assess regional involvement, and echocardiography to detect concurrent cardiac hemangiosarcoma, which has been reported in a significant proportion of dogs with splenic disease [3,10,14]. Even when comprehensive clinical staging is performed, micrometastatic disease is presumed to be present in most cases at the time of diagnosis, contributing to disease progression despite complete macroscopic tumor removal [14,17].

## 4. Prognostic Factors and Clinical Staging

Clinical staging and prognostic assessment play a central role in the management of dogs with splenic hemangiosarcoma. Tumor staging is most commonly performed using the TNM system of the World Health Organization, which considers the anatomical extent of the primary tumor, regional lymph node involvement, and the presence of distant metastases [16]. Based on this system, splenic hemangiosarcoma is generally classified into three clinical stages. Stage I refers to tumors confined to the spleen without evidence of rupture or metastasis; stage II includes tumors associated with splenic rupture and hemoperitoneum and/or regional lymph node involvement; and stage III is defined by the presence of distant metastases [16,18].

Clinical stage at diagnosis is consistently reported as one of the most important prognostic factors in dogs with splenic hemangiosarcoma [16,17,18,19,20]. Dogs diagnosed at stage I generally have longer survival times than those presenting with stage II or stage III disease [19,20]. Several retrospective studies have demonstrated that advanced disease stage is associated with shorter median survival time and shorter disease-free intervals, reflecting the higher tumor burden and increased likelihood of metastatic spread [17,20,29]. In particular, dogs with stage III disease typically have the poorest prognosis, largely due to the persistence of macroscopic metastatic disease even after splenectomy [18,29].

The prognostic distinction between stage I and stage II disease is less clearly defined. While both stages may be free of detectable distant metastases at the time of diagnosis, stage II disease includes dogs with splenic rupture and hemoperitoneum as well as those with large, non-ruptured splenic tumors. Splenic rupture with hemoperitoneum has traditionally been considered a factor that may increase the risk of tumor cell dissemination within the abdominal cavity [18]. Tumor rupture may facilitate local implantation of neoplastic cells on serosal surfaces, potentially contributing to peritoneal metastasis and more rapid disease progression. In addition, dogs classified as stage II may already harbor subclinical metastatic disease that is not detectable using conventional staging methods [14,18].

Histopathological features have also been investigated as prognostic indicators in splenic hemangiosarcoma. Tumor grade, based on criteria such as degree of differentiation, nuclear pleomorphism, extent of necrosis, and mitotic index, has been associated with survival outcomes in several studies [13,16,18]. Poorly differentiated tumors with marked pleomorphism, high mitotic activity, and extensive necrosis are generally associated with a more aggressive clinical course and shorter survival times [13,16]. Nevertheless, the prognostic value of histological grading is not uniformly reported across studies, and its independent contribution relative to clinical stage remains uncertain.

Additional clinicopathological parameters have been evaluated for their potential prognostic significance. Thrombocytopenia at diagnosis has been associated with shorter survival times in dogs with splenic hemangiosarcoma and may reflect advanced disease or increased tumor-associated consumption of platelets [29]. Anemia, although commonly present, appears to be more closely related to acute hemorrhage than to long-term prognosis [28]. Age, breed, and sex have also been examined as prognostic factors, but results have been inconsistent, and their clinical relevance remains unclear [5,14].

It is also important to recognize that prognostic factors in splenic hemangiosarcoma are often interrelated and should be interpreted within a broader clinical context. Clinical stage, histopathological features, and clinicopathological abnormalities may coexist and reflect different aspects of tumor burden and biological aggressiveness [13,18,29]. Consequently, reliance on a single prognostic parameter may be insufficient to accurately predict outcome in individual patients. This multifactorial nature of prognosis is associated with inherent uncertainty in outcome prediction in dogs with splenic hemangiosarcoma and with the limitations of current prognostic models in routine clinical practice [13,14,16,18].

Despite advancements in staging and therapeutic protocols, there is currently a lack of reliable prognostic biomarkers or clinical factors capable of identifying the subset of long-term survivors. While the overall prognosis for canine hemangiosarcoma remains poor, approximately 10% of patients exhibit exceptional survival times exceeding one year; unfortunately, it remains impossible to prospectively distinguish these individuals at the time of diagnosis [14,17]. The identification of such prognostic indicators would be of paramount importance, as it would provide owners with more accurate expectations and significantly aid in their decision-making process regarding aggressive treatment options.

Interpretation of prognostic data in splenic hemangiosarcoma must therefore be approached with caution. Most available studies are retrospective in design [18,19,24,27,31], with heterogeneous inclusion criteria and variable staging procedures. Differences in histopathological grading approaches and case selection further limit direct comparison between studies [13,14,18]. These methodological factors likely contribute to variability in reported survival times across studies [16,18,20,24] and may partially explain discrepancies in the prognostic significance attributed to individual clinicopathological variables.

## 5. Therapeutic Approaches

### 5.1. Surgery

**Figure 3:**
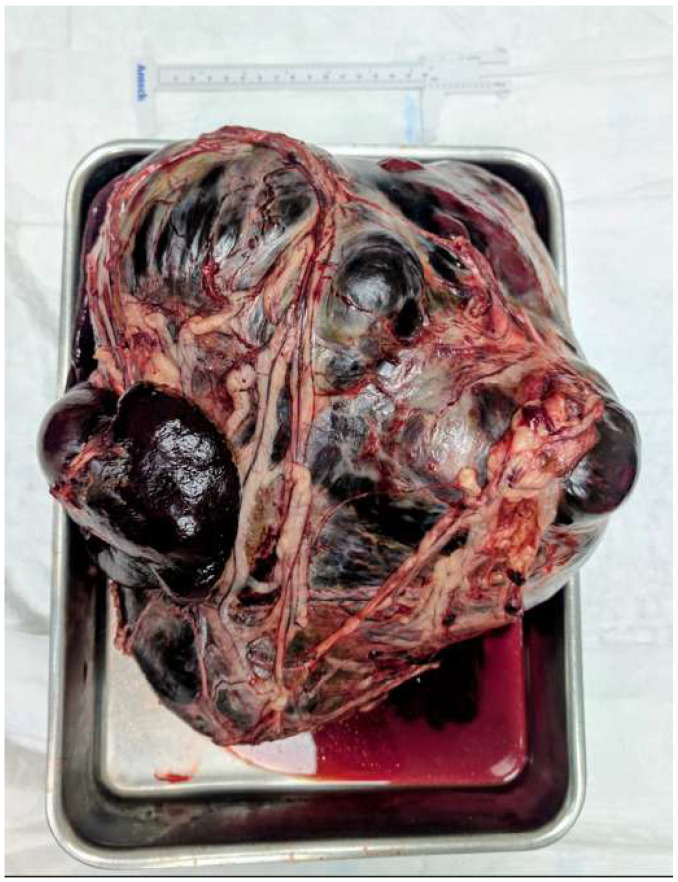
Post-operative gross appearance of canine splenic hemangiosarcoma with massive hematoma. The spleen is markedly enlarged and distorted by a very large, multilobulated splenic mass with extensive hemorrhage. The lesion shows mixed coloration with dark red areas consistent with blood-filled cavities/hematoma and multifocal hemorrhagic and congested regions beneath the splenic capsule, reflecting the friable, highly vascular nature of the tumor (image courtesy of Onevet Porto Veterinary Hospital).

Surgical removal of the spleen remains the cornerstone of initial treatment for dogs diagnosed with splenic hemangiosarcoma. Splenectomy is primarily indicated to control life-threatening hemorrhage associated with splenic rupture and hemoperitoneum and to remove macroscopic disease [14,16,21]. In many cases, surgery is performed on an emergency basis, often before a definitive diagnosis is established, due to the acute clinical presentation of affected dogs (Figure 3).

Despite its essential role in stabilizing the patient, splenectomy alone is considered a largely palliative procedure in the context of splenic hemangiosarcoma. Median survival times following surgery as a sole treatment are consistently short, reflecting the high likelihood of metastatic disease at the time of diagnosis [14,16]. During exploratory laparotomy, careful inspection of the abdominal cavity is recommended, and any suspicious lesions in organs such as the liver or omentum should be sampled for histopathological evaluation, as concurrent metastatic disease is common [14].

Interestingly, some studies have reported longer survival times in older dogs undergoing splenectomy for splenic hemangiosarcoma compared with younger animals, although the biological basis for this observation remains unclear [5]. Nevertheless, surgery alone does not address micrometastatic disease and is insufficient to significantly alter the overall disease course in most cases [14]. 

### 5.2. Conventional Chemotherapy

Given the high metastatic rate associated with splenic hemangiosarcoma, adjuvant chemotherapy has been widely employed following splenectomy in an attempt to delay disease progression and improve survival outcome (Table 1). Table 1 provides a compilation of selected studies evaluating various chemotherapeutic protocols and their respective clinical outcomes [16,17,18,19,20,21,31,32,33,34,35,36,37,38,39,40,41]. Doxorubicin-based protocols are the most commonly used and are generally considered the standard of care for dogs without overt metastatic disease at the time of diagnosis [5,14].

Doxorubicin may be administered as monotherapy or in combination with other chemotherapeutic agents. Several retrospective and prospective clinical studies have evaluated the efficacy of doxorubicin as a single agent following splenectomy. Ogilvie et al. (1996) [16] reported a median survival time of approximately 4–6 months in dogs treated with surgery followed by doxorubicin, representing a significant improvement compared with surgery alone, although long-term survival remained uncommon [16]. Subsequent studies confirmed these findings, consistently demonstrating modest but reproducible survival benefits with adjuvant doxorubicin, regardless of minor protocol variations [19,20].

Combination chemotherapy protocols have been investigated with the aim of improving therapeutic efficacy by targeting different cellular pathways. These include combinations of doxorubicin with cyclophosphamide (AC), vincristine (VAC), or dacarbazine (DAV) [14,19,21,31]. The AC protocol has been evaluated in several retrospective series, showing median survival times that were numerically longer than those reported with doxorubicin alone in some cohorts, although differences were not consistently statistically significant [20,21]. Importantly, these studies often included heterogeneous populations in terms of clinical stage, which may partly explain the variability in reported outcomes.

The combination of doxorubicin and dacarbazine has received particular attention. In a comparative study by Finotello et al. (2017) [21], dogs with visceral hemangiosarcoma treated with doxorubicin and dacarbazine demonstrated prolonged median survival time and delayed development of metastatic disease when compared with dogs receiving doxorubicin and cyclophosphamide, with acceptable toxicity profiles [21].

More aggressive multi-agent protocols, such as DAV, have been explored primarily in dogs with advanced-stage disease. Dervisis et al. (2011) [31] evaluated DAV in dogs with stage II and III hemangiosarcoma and reported median survival times exceeding those historically associated with surgery alone; however, treatment-related toxicity, particularly hematologic and gastrointestinal adverse effects, led to protocol discontinuation in nearly 20% of patients [31].

Alternative anthracyclines and platinum-based agents have also been investigated. Epirubicin, a stereoisomer of doxorubicin, has shown comparable efficacy in the adjuvant setting, with Kim et al. (2007) reporting median survival times like those achieved with conventional doxorubicin-based protocols, while offering the advantage of reduced cardiotoxicity [40]. Carboplatin has likewise been evaluated as an alternative agent, particularly in dogs at risk of anthracycline-induced cardiotoxicity, with reported survival outcomes broadly comparable to doxorubicin in retrospective analyses [32].

Overall, although adjuvant chemotherapy clearly prolongs survival compared with splenectomy alone, the magnitude of benefit achieved with conventional chemotherapy remains limited, and durable disease control is rarely observed. Across studies, substantial variability in survival outcomes persists, reflecting differences in study design, case selection, clinical stage distribution, and underlying tumor biology, rather than consistent superiority of any single chemotherapeutic protocol [14,17,18].

### 5.3. Metronomic Chemotherapy

Metronomic chemotherapy, typically involving the continuous administration of low-dose chemotherapeutic agents, has been explored as an alternative or adjunctive treatment strategy in dogs with splenic hemangiosarcoma, based on its proposed antiangiogenic and immunomodulatory effects [18,19]. Cyclophosphamide is the most commonly used agent in metronomic protocols, frequently administered in combination with nonsteroidal anti-inflammatory drugs (NSAIDs) with putative antiangiogenic properties, such as piroxicam, firocoxib or deracoxib [19,42].

One of the earliest clinical evaluations of metronomic chemotherapy in splenic hemangiosarcoma was reported by Lana et al. (2007) [33], who assessed continuous low-dose oral cyclophosphamide as adjuvant therapy following splenectomy. Although the study suggested acceptable tolerability and a potential delay in disease progression, median survival times were not markedly superior to those reported with conventional doxorubicin-based protocols [33].

Subsequent retrospective studies evaluated metronomic chemotherapy either as a standalone treatment or in combination with conventional chemotherapy. Wendelburg et al. (2015) [18] compared outcomes in dogs treated with splenectomy alone, splenectomy followed by doxorubicin, and splenectomy followed by doxorubicin combined with metronomic cyclophosphamide. While dogs receiving adjuvant chemotherapy, had improved survival compared with surgery alone, the addition of metronomic chemotherapy did not confer a significant survival advantage over conventional chemotherapy [18].

More recent analyses have reinforced these findings. Matsuyama et al. (2017) [17] evaluated adjuvant doxorubicin with or without metronomic cyclophosphamide and found no significant improvement in median survival time or disease-free interval associated with the metronomic component [17]. Similarly, Alexander et al. (2019) [22] reported that the addition of metronomic chemotherapy did not improve outcomes in dogs with splenic hemangiosarcoma, despite theoretical benefits related to angiogenesis inhibition [22].

A retrospective comparison by Treggiari et al. (2020) [24] further examined first-line anthracycline-based protocols versus metronomic-based chemotherapy in dogs with stage I and II splenic hemangiosarcoma. The study did not identify a significant survival advantage for metronomic protocols and reported substantial overlap in outcomes between treatment groups, underscoring the difficulty in defining a clear therapeutic niche for metronomic chemotherapy in this disease [24]. Overall, metronomic chemotherapy represents a biologically appealing approach, particularly considering the vascular nature of hemangiosarcoma. Nevertheless, clinical studies have shown heterogeneous results regarding survival outcomes, with metronomic chemotherapy failing to demonstrate a consistent survival advantage over conventional chemotherapy protocols in dogs with splenic hemangiosarcoma [17,18,22,24]. Table 2 summarizes median survival times reported in dogs with splenic hemangiosarcoma treated with metronomic chemotherapy-based protocols.

### 5.4. Immunotherapy and Other Therapeutic Strategies

Immunotherapy has been explored as an adjunctive treatment strategy for canine splenic hemangiosarcoma, based on the rationale of enhancing host antitumor immune responses while potentially reducing the toxicity associated with conventional chemotherapy [43]. Among immunomodulatory agents, Muramyl Tripeptide Phosphatidylethanolamine (MTP-PE) has been investigated for its ability to activate the mononuclear phagocytic system, leading to increased production of cytokines and tumor necrosis factors and, consequently, enhanced antitumor activity [10]. When used in combination with conventional chemotherapy, MTP-PE has been associated with increases in median survival time in selected studies, although outcomes remain variable [10].

MTP-PE is encapsulated in liposomes, enhancing cellular endocytosis, increasing tumor exposure to the immunomodulator, and prolonging its blood concentration [10,44]. This liposomal formulation has also been reported to reduce the cardiotoxicity associated with doxorubicin, representing a potential advantage when these agents are used in combination protocols [10].

Vaccination-based strategies have likewise been investigated in canine hemangiosarcoma, generally as adjuncts to conventional chemotherapy. U’Ren et al. (2007) [45] evaluated an intraperitoneal vaccine derived from hemangiosarcoma cell line lysates in a rat model and reported prolonged median survival time compared with chemotherapy alone, with good tolerability [45]. In canine patients, Konduri et al. (2019) [46] assessed an autologous dendritic cell vaccine, while Lucroy et al. (2020) [43] evaluated immunotherapeutic approaches in dogs with hemangiosarcoma, both reporting longer survival times compared with splenectomy alone [23,46]. However, in the latter study, median survival times were similar between dogs receiving conventional chemotherapy and those treated with immunotherapy, underscoring the difficulty in demonstrating superiority over established cytotoxic protocols. Across studies, vaccination strategies were generally well tolerated and capable of inducing humoral immune responses against neoplastic cells.

Angiogenesis represents a key mechanism underlying tumor progression and metastatic dissemination in hemangiosarcoma. Angiogenesis inhibitors, such as interferon and thalidomide, have been proposed as therapeutic agents based on their ability to limit neovascularization and metastatic growth [10]. Unlike conventional cytotoxic chemotherapy, these agents are not typically associated with gastrointestinal toxicity or bone marrow suppression, contributing to a favorable safety profile. However, angiogenesis inhibitors do not exert direct cytotoxic effects on the primary tumor and are therefore unlikely to be effective as monotherapy, supporting their use only in combination with other treatment modalities [10,47].

Matrix metalloproteinases (MMPs) play a central role in tumor invasion and metastasis by degrading extracellular matrix components, including collagen and glycoproteins, thereby facilitating neoplastic cell migration into lymphatic and blood vessels. Increased MMP activity has been documented in dogs with neoplasia compared with healthy controls, suggesting a role in tumor progression [10]. Minocycline, a tetracycline-derived antibiotic, has been investigated for its inhibitory effects on both MMP activity and angiogenesis. In a study by Sorenmo et al. (2000) [48], minocycline was added to a standard chemotherapy protocol consisting of doxorubicin and cyclophosphamide; however, no significant survival advantage was observed compared with conventional chemotherapy alone [48]. Most dogs in that study succumbed to tumor-related causes, with post-mortem examination revealing widespread metastatic disease. Possible explanations for the lack of therapeutic benefit include suboptimal dosing, activation of alternative pro-angiogenic pathways, or a limited role of collagenases in the progression of hemangiosarcoma [48].

Targeted therapies have also been evaluated in canine hemangiosarcoma. Expression of tyrosine kinase family receptors has been documented in neoplastic cells, and agents such as masitinib, imatinib, and dasatinib have demonstrated antiproliferative and pro-apoptotic effects in hemangiosarcoma cell lines. However, achieving therapeutically effective drug concentrations in vivo without inducing unacceptable toxicity remains challenging. Toceranib phosphate, which inhibits platelet-derived growth factor receptors and vascular endothelial growth factor receptors, has shown in vitro activity against several tumor types. Nevertheless, its use in dogs with stage I and II splenic hemangiosarcoma, either in combination with doxorubicin or following conventional chemotherapy, did not result in statistically significant improvements in disease-free interval or median survival time [14,23].

Overall, while immunotherapy, angiogenesis inhibition, matrix metalloproteinase inhibition, and targeted therapies represent biologically appealing approaches, current clinical evidence does not support their use as standalone alternatives to conventional chemotherapy in canine splenic hemangiosarcoma. A multimodal therapeutic approach therefore remains the most rational strategy, although the optimal integration of these emerging modalities into routine clinical practice has yet to be clearly defined.

Importantly, the interpretation of therapeutic efficacy across studies requires careful consideration of methodological limitations. Available data derive predominantly from retrospective analyses [18,19,24,27,31], with heterogeneous inclusion criteria and variable stage distribution [13,14,24]. In addition, differences in chemotherapy protocols, including dose intensity, treatment duration, and combination strategies, further limit direct comparison between studies [16,20,21,24]. The absence of standardized therapeutic algorithms and the limited number of prospective randomized clinical trials significantly restrict the ability to establish the superiority of one protocol over another. Consequently, reported differences in median survival times may reflect study design variability rather than true biological differences in treatment response [16,18,20,24].

## 6. Prognosis

Canine splenic hemangiosarcoma is associated with a poor prognosis, reflecting its aggressive biological behavior, high metastatic potential, and limited response to currently available therapeutic strategies. Dogs treated with splenectomy alone generally have short median survival times, most commonly ranging from a few weeks to a few months, largely due to the presence of undetected metastatic disease at the time of diagnosis [14,16,18].

The addition of adjuvant chemotherapy, particularly doxorubicin-based protocols, has been shown to prolong median survival time when compared with surgery alone; however, the magnitude of this benefit remains limited [14,16,17,18,19,20]. Reported median survival times for dogs receiving surgery followed by chemotherapy typically range from approximately four to seven months, with long-term survival being uncommon [14,17,19]. Even with multimodal treatment, one-year survival rates remain low, generally reported to be below 10% [14].

Clinical stage at diagnosis remains one of the most consistent prognostic indicators. Dogs diagnosed with stage I disease tend to have longer survival times than those presenting with stage II or stage III disease, while dogs with overt metastatic disease at diagnosis have the poorest outcomes [16,17,18,19,20,29]. Tumor rupture and hemoperitoneum, which characterize stage II disease, have been associated with shorter survival in some studies, although the prognostic distinction between stage I and stage II remains variable across reports [18].

Histopathological features, including tumor grade, mitotic index, degree of differentiation, and extent of necrosis, have also been evaluated as prognostic factors, with higher-grade tumors generally associated with shorter survival times [13,16,18]. However, variability in grading systems and study design limits the ability to apply histological grading consistently in clinical practice.

Additional factors such as thrombocytopenia at diagnosis have been associated with poorer outcomes and may reflect advanced disease or increased tumor burden [29]. Other variables, including age, breed, and sex, have been investigated, but their prognostic significance remains inconsistent and is not uniformly supported across studies [5,14].

Despite advances in diagnostic imaging, surgical management, and adjuvant therapies, overall prognosis for dogs with splenic hemangiosarcoma has not substantially improved over time. Early metastatic dissemination and limitations in current staging methods continue to constrain long-term disease control, even in cases diagnosed at apparently localized stages [14,17].

## 7. Future Directions

Despite decades of clinical research, therapeutic progress in canine splenic hemangiosarcoma has been limited. Future advances will likely depend on improved biological stratification and methodological standardization. The development of robust prognostic and predictive biomarkers capable of identifying high-risk patients and potential long-term survivors remains a priority. Molecular profiling approaches may facilitate subclassification of splenic hemangiosarcoma into biologically distinct entities, potentially enabling more personalized therapeutic strategies.

In addition, the design of prospective, multicentric clinical trials with standardized staging criteria and treatment protocols is essential to generate higher-quality evidence and allow meaningful comparison between therapeutic approaches. Integration of emerging modalities, including targeted therapies and immunomodulatory strategies, should be guided by biologically rational combinations rather than empirical escalation of cytotoxic regimens.

A deeper understanding of tumor–microenvironment interactions and mechanisms of therapeutic resistance may further support the development of innovative treatment paradigms. Ultimately, progress in this field will require collaborative efforts that bridge molecular research and clinical oncology, with the goal of translating biological insights into measurable improvements in survival and quality of life for affected dogs.

## 8. Conclusions

Canine splenic hemangiosarcoma remains one of the most aggressive and clinically challenging neoplasms in dogs. Its biological behavior, characterized by rapid growth, vascular instability, and early metastatic dissemination, underlies the high mortality associated with this disease. The frequent absence of clinical signs until acute presentation, often due to splenic rupture and hemoperitoneum, limits opportunities for early diagnosis and comprehensive staging.

Clinical staging continues to be a relevant prognostic tool, yet its ability to accurately predict individual outcomes is constrained by the high prevalence of occult metastatic disease at the time of diagnosis. Similarly, histopathological and clinicopathological parameters may offer prognostic insight but do not fully capture the biological complexity of this tumor.

Overall, the persistent poor prognosis associated with canine splenic hemangiosarcoma reflects both its inherent biological aggressiveness and the limitations of currently available diagnostic and therapeutic options. Continued efforts to refine prognostic assessment and therapeutic approaches are essential to improve clinical outcomes in affected dogs.

## Figures and Tables

**Table 1:** Reported median survival times following splenectomy with or without adjuvant chemotherapy in dogs with hemangiosarcoma).

Treatment	Study (Author, Year)	Study Design and Sample Size (Total)	Tumor Type	Stage Distribution	Number of Animals Included in the Protocol	Median Survival (Days) *
**Splenectomy alone**	Wendelburg et al., 2015 [18]	Retrospective (*n* = 208)	SH	Mixed stages (I + II + III)	154	48
	Batschinski et al., 2018 [19]	Retrospective (*n* = 37)	H	Stages I + III	23	66
**Doxorubicin**	Ogilvie et al., 1996 [16]	Prospective (*n* = 46)	H	Stages I + III	46	172
	Batschinski et al., 2018 [19]	Retrospective (*n* = 37)	H	Stages I + III	14	274
	Sorenmo et al., 2004 [20]	Prospective (*n* = 20)	H	Stage I	5	257
				Stage II	9	186
				Stage III	6	87
	Matsuyama et al., 2017 [17]	Retrospective (*n* = 33)	SH	Mixed stages (I + II + III)	15	143
	Faulhaber et al., 2021 [32]	Retrospective (*n* = 40)	SH	Stages I + II	22	139
	Lana et al., 2007 [33]	Retrospective (*n* = 31)	SH	Stage II	24	133
**Doxorubicin + Cyclophosphamide (AC)**	Sorenmo et al., 1993 [34]	Prospective (*n* = 16)	H	Mixed stages (I + II + III)	16	202
	Finotello et al., 2017 [21]	Prospective (*n* = 27)	H	Stages II + III	18	142
**Doxorubicin + Cyclophosphamide + Vincristine** **(VAC)**	Alvarez et al., 2013 [35]	Retrospective (*n* = 67)	SH	Stages I + II	42	189
				Stage III	25	195
	Hammer et al., 1991 [36]	Prospective (*n* = 15)	SH	Mixed stages (I + II + III)	15	172
**Doxorubicin + Vincristine** **+ Dacarbazine** **(DAV)**	Dervisis et al., 2011 [31]	Prospective (*n* = 24)	H	Mixed stages (I + II + III)	24	125
**Doxorubicin + Dacarbazine** **(ADTIC)**	Finotello et al., 2017 [21]	Prospective (*n* = 27)	H	Stages II + III	9	>550
**Doxorubicin + Deracoxib**	Kahn et al., 2013 [37]	Prospective (*n* = 21)	SH	Stage I	3	239
				Stage II	7	120
				Stage III	11	149
				Mixed stages (I + II + III)	21	150
**Carboplatin-based protocol**	Faulhaber et al., 2021 [32]	Retrospective (*n* = 40)	SH	Stage I	8	341
				Stage II	10	97.5
				Stages I + II	18	160
**Ifosfamide**	Rassnick et al., 2000 [38]	Retrospective (*n* = 72)	SH	Stages I + II	6	147–206
**Ifosfamide + Doxorubicin**	Payne et al., 2003 [39]	Retrospective (*n* = 39)	SH	Stages I + III	27	149
**Epirubicin**	Kim et al., 2007 [40]	Retrospective (*n* = 59)	SH	Stage I	7	983
				Stage II	3	98
				Stage III	8	135
				Mixed stages (I + II + III)	18	144
**Thalidomide**	Bray et al., 2008 [41]	Prospective (*n* = 15)	SH	Stage II	10	303
				Stage III	5	40
				Stage II + III	15	172

* Refers to the median survival time of the animals in a given protocol; H: Canine Hemangiosarcoma, except cutaneous; SH: Splenic Hemangiosarcoma.

**Table 2:** Median survival associated with the administration of metronomic chemotherapy in the treatment of splenic hemangiosarcoma.

Treatment	Study (Author, Year)	Study Design and Sample Size (Total)	Stage Distribution	Number of Animals Included in the Protocol	Median Survival (Days) *
**Conventional** **Chemotherapy**	Lana et al., 2007 [33]	Retrospective (*n* = 31)	Stage II	24	133
	Matsuyama et al., 2017 [17]	Retrospective (*n* = 33)	Mixed stages (I + II + III)	15	143
	Wendelburg et al., 2015 [18]	Retrospective (*n* = 208)	Mixed stages (I + II + III)	28	90
	Alexander et al., 2019 [22]	Retrospective (*n* = 51)	Mixed stages (I + II + III)	39	180
	Treggiari et al., 2020 [24]	Retrospective (*n* = 109)	Stages I + II	75	154
**Metronomic** **therapy**	Wendelburg et al., 2015 [18]	Retrospective (*n* = 208)	Mixed stages (I + II + III)	13	102
	Treggiari et al., 2020 [24]	Retrospective (*n* = 109)	Stages I + II	34	225
	Lana et al., 2007 [33]	Retrospective (*n* = 31)	Stage II	9	178
**Conventional Chemotherapy + Metronomic Chemotherapy**	Matsuyama et al., 2017 [17]	Retrospective (*n* = 33)	Mixed stages (I + II + III)	18	186
	Wendelburg et al., 2015 [18]	Retrospective (*n* = 208)	Mixed stages (I + II + III)	13	129
	Alexander et al., 2019 [22]	Retrospective (*n* = 51)	Mixed stages (I + II + III)	22	212
	Treggiari et al., 2020 [24]	Retrospective (*n* = 109)	Stages I + II	23	338

* Refers to the median survival time of the animals in a given protocol.

## Data Availability

No new data were created or analyzed in this study. Data sharing is not applicable to this article.
